# Nature inspired method for noninvasive fetal ECG extraction

**DOI:** 10.1038/s41598-022-24733-1

**Published:** 2022-11-23

**Authors:** Akshaya Raj, Jindrich Brablik, Radana Kahankova, Rene Jaros, Katerina Barnova, Vaclav Snasel, Seyedali Mirjalili, Radek Martinek

**Affiliations:** 1 Department of Cybernetics and Biomedical Engineering, Faculty of Electrical Engineering and Computer Science, VSB-Technical University of Ostrava, 17. listopadu, Ostrava, 708 00 Czechia; 2 Department of Computer Science, Faculty of Electrical Engineering and Computer Science, VSB-Technical University of Ostrava, 17. listopadu, Ostrava, 708 00 Czechia; 3 Centre for Artificial Intelligence Research and Optimisation, Torrens University Australia, 90 Bowen Terrace, Brisbane, QLD 4006 Australia

**Keywords:** Biomedical engineering, Cardiology, Medical research, Data processing

## Abstract

This paper introduces a novel algorithm for effective and accurate extraction of non-invasive fetal electrocardiogram (NI-fECG). In NI-fECG based monitoring, the useful signal is measured along with other signals generated by the pregnant women’s body, especially maternal electrocardiogram (mECG). These signals are more distinct in magnitude and overlap in time and frequency domains, making the fECG extraction extremely challenging. The proposed extraction method combines the Grey wolf algorithm (GWO) with sequential analysis (SA). This innovative combination, forming the GWO-SA method, optimises the parameters required to create a template that matches the mECG, which leads to an accurate elimination of the said signal from the input composite signal. The extraction system was tested on two databases consisting of real signals, namely, Labour and Pregnancy. The databases used to test the algorithms are available on a server at the generalist repositories (figshare) integrated with Matonia et al. (Sci Data 7(1):1–14, 2020). The results show that the proposed method extracts the fetal ECG signal with an outstanding efficacy. The efficacy of the results was evaluated based on accurate detection of the fQRS complexes. The parameters used to evaluate are as follows: accuracy (ACC), sensitivity (SE), positive predictive value (PPV), and F1 score. Due to the stochastic nature of the GWO algorithm, ten individual runs were performed for each record in the two databases to assure stability as well as repeatability. Using these parameters, for the Labour dataset, we achieved an average ACC of 94.60%, F1 of 96.82%, SE of 97.49%, and PPV of 98.96%. For the Pregnancy database, we achieved an average ACC of 95.66%, F1 of 97.44%, SE of 98.07%, and PPV of 97.44%. The obtained results show that the fHR related parameters were determined accurately for most of the records, outperforming the other state-of-the-art approaches. The poorer quality of certain signals have caused deviation from the estimated fHR for certain records in the databases. The proposed algorithm is compared with certain well established algorithms, and has proven to be accurate in its fECG extractions.

## Introduction

The fetal electrocardiogram (fECG) reveals the state of the fetus. It contains clinically important information regarding fetal well being and can be used to identify possible pathological states, such as myocardial ischemia, intrapartum hypoxia, or metabolic acidosis. These life threatening conditions manifest themselves as changes in the frequency but also the morphology of the fECG signal, where the latter cannot be accessed using the conventional means of monitoring^1^. Non-invasive fetal electrocardiography (NI-fECG) is one of the most promising methods that has shown reliable results for long term monitoring of fetal heart rate (fHR)^2^. This technique records electrical potentials using the electrodes placed on the maternal abdomen. The signals measured are a mixture of both maternal and fetal components and a significant amount of noise (such as noise from the maternal muscle and organ activity) overlapping in time and frequency domains. Moreover, the magnitude of the fetal component is small compared to the magnitudes of the rest of the signals (especially maternal component), which makes the accurate extraction of clinically relevant features challenging^3^. However, the development of advanced signal processing methods makes the fECG extraction possible, and thus, this method could become a useful tool in the clinical practice of obstetrics and gynaecology for continuous and non-invasive fetal monitoring.

Many different methods have been introduced for fECG signal extraction from abdominal ECG signals^1,4–6^. Adaptive filters are known to produce promising results^1^. However, the efficacy of the adaptive extraction systems strongly depends on the quality of the input signals, particularly by the reference input (i.e. thoracic maternal ECG), that may be affected by the maternal motion, breathing activity or unsuitable contact of the electrode with the skin at the thoracic area. Therefore, it could be quite complicated to maintain the signal’s high quality in the clinical practice. For this reason, several authors^6–8^ used an alternative approach to adaptive extraction, where the reference maternal signal is estimated directly from the abdominal inputs by using, for example, blind source separation, such as Independent component analysis (ICA)^9^ or Principal Component Analysis (PCA)^10,11^. Both PCA and ICA methods decompose the input signal into its source components. These two methods, though useful in extraction of fECG through the removal of artifacts and noise, have some drawbacks. With ICA, though higher inputs leads to higher precision in results^1^, it also leads to higher dimensionality and higher computational complexity. In most of the cases, the principal component found in abdominal input corresponds to the maternal component^9^. The performance of the PCA method decreases with a lower power ratio between the weak source (fECG) and the strong source (mECG)^9^. Therefore, its use is ideal for cases where maternal component is dominant in the abdominal mixture.

Over the past few years, a lot of stochastic optimisation techniques have emerged. They can be classified in several ways: gradient, evolutionary, swarm based and many more. Amongst the most commonly known gradient methods are the Least Mean Square (LMS) and Recursive Least Square (RLS) algorithms. Genetic algorithm is the most popular evolutionary based optimisation algorithm. Swarm based algorithms use the social behaviour of animals. A popular algorithm under swarm based algorithm is Particle Swarm Optimisation (PSO)^12^. Some more optimisation methods are: Artificial Bee Colony (ABC)^13^, Cuckoo search algorithm^14^, Firefly algorithm^15^, Dolphin Partner Optimization algorithm^16^. These algorithms optimise the cost function using the hunting and search patterns of animals. One such algorithm, which is also inspired by nature, is Grey Wolf Optimiser (GWO) proposed by Seyedali Mirajalili et al.^17^.

In this paper, a novel hybrid algorithm is presented that uses GWO method with a non-blind method called Sequential Analysis (SA) to extract the fECG. Using a nature-based algorithm along with an already existing algorithm to optimise one or more parameters is expanding in various fields. The field of fECG extraction is an area where there is still vast scope to conduct extensive research related to optimisation using meta-heuristics to observe how well it performs. The SA uses a priori information about the signal to extract the fECG. The GWO proficiently utilises the exploration/exploitation abilities to optimise the variables in SA that would increase the accuracy of the results. Our method is evaluated on two different databases, namely, Labour and Pregnancy, which were obtained from a publicly available data set (see “Datasets”)^18^. The use of GWO with a non-blind method, such as SA, is a novel concept in the field of fECG extraction. Also, the algorithm is tested on *real* signals and does not rely on the simplicity of the synthetic data. As the method is stochastic, the experiment on each signal is conducted ten individual times to assure repeatability.

The above brief literature review shows that the current gap is in the lack of techniques that would ensure precise fECG extraction for a universal use. Although adaptive filters are potentially highly accurate, their performance depends on the system settings that are not adjustable according to the variable conditions that appear in clinical practice. This motivated our attempt to employ a recently proposed algorithm called GWO for extracting non-invasive fECG. This approach could provide a highly reliable solution for both non-invasive fetal heart rate monitoring or further morphological analysis.

The rest of the paper is organized as follows: “Introduction” presents the GWO algorithm and literature review of ECG extraction. “Materials and methods2” introduces the proposed algorithm. Experimental setups are provided in “Sequential analysis with grey Wolf optimisation”. “Results” presents the results from the experiments. Finally, “Discussion” presents, discusses, and analyses results. “Conclusion” concludes the work and suggests future directions.

## Materials and methods

This section presents the two algorithms that are integrated. First, the GWO algorithm is presented, and then, the details of the SA method is given. This section also presents the datasets used as well as the evaluation protocol. We confirm that all methods were performed in accordance with the relevant guidelines and regulations.

### Grey Wolf optimiser

**Figure 1:**
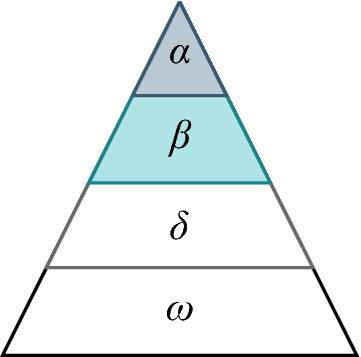
Grey Wolf hierarchy.

Grey wolves are canines that belong to the Canidae family. The wolves live and hunt in packs, and are highly intelligent animals with a strong sense of social hierarchy. The GWO method draws from the social hierarchy and the hunting mechanism from the operation methods of a pack of wolves, see Fig. 1. The parameters that lead the method are called alpha ($\alpha$), beta ($\beta$), delta ($\delta$) and in some cases an additional parameter is added, named omega ($\omega$).

According to the mathematical model, $\alpha$ is considered the best solution. $\beta$ and $\delta$ are considered second best and third best solutions, respectively. Muro et al.^19^ gives us the three rules that the packs follows to catch the prey or in case of optimisation, get a global minima.

1. Hunting and tracking the prey
2. Chasing and encircling the prey
3. Attacking the prey

These phases are implemented within the GWO to perform optimisation. The following equations are the mathematical realisation of the above mentioned concepts.

The following two equations are the proposed mathematical model of the *encircling* behaviour of the wolves^17^:

1 $$\begin{aligned} \vec {D}=\left| \vec {C} \cdot \overrightarrow{X_{p}}(t)-\vec {X}(t)\right| \end{aligned}$$

2 $$\begin{aligned} \vec {X}(t+1)=\overrightarrow{X_{p}}(t)-\vec {A} \cdot \vec {D} \end{aligned}$$

where *t* represents the current iteration, *A* and *C* represents the coefficient vectors, $\overrightarrow{X_{p}}$ represents the position vector of the prey and $\vec {X}$ represents the position vector of a grey wolf.

The coefficient vector *A* and *C* are calculated using the formulae below:

3 $$\begin{aligned} \vec {A}=2 \vec {a} \cdot \vec {r}_{1}-\vec {a}, \end{aligned}$$

4 $$\begin{aligned} \vec {C}=2 \cdot \vec {r}_{2}, \end{aligned}$$

where $r_1$,$r_2$ are random vectors in [0, 1] and the values of $\vec {a}$ linearly decrease from 2 to 0 during the iteration using the equation below:

5 $$\begin{aligned} a(t)=2-(2 \times t) / \text{ MaxIter } . \end{aligned}$$

After the encircling phase, the wolves enter the hunting phase. The wolves now have a better knowledge of the location of the prey (global minima). The alpha (best solution) leads the hunt and the rest of the wolves are to follow. The positions of the three best solutions are saved and that leads to every other search agent, a.k.a wolves, updating their positions accordingly. The following equations are mathematical model of the *hunting* behaviour:

6 $$\begin{aligned}{} & {} \begin{array}{c} \vec {D}_{\alpha }=\left| \vec {C}_{1} \cdot \vec {X}_{\alpha }-\vec {X}\right| ,\\ \vec {D}_{\beta }=\left| \vec {C}_{2} \cdot \vec {X}_{\beta }-\vec {X}\right| ,\\ \vec {D}_{\delta }=\left| \vec {C}_{3} \cdot \vec {X}_{\delta }-\vec {X}\right| .\\ \end{array} \end{aligned}$$

7 $$\begin{aligned}{} & {} \begin{array}{c} \vec {X}_{1}=\vec {X}_{\alpha }-\vec {A}_{1} \cdot \left( \vec {D}_{\alpha }\right) ,\\ \vec {X}_{2}=\vec {X}_{\beta }-\vec {A}_{2} \cdot \left( \vec {D}_{\beta }\right) ,\\ \vec {X}_{3}=\vec {X}_{\delta }-\vec {A}_{3} \cdot \left( \vec {D}_{\delta }\right) .\\ \end{array} \end{aligned}$$

8 $$\begin{aligned}{} & {} \vec {X}(t+1)=\frac{\vec {X}_{1}+\vec {X}_{2}+\vec {X}_{3}}{3}. \end{aligned}$$

From this point on, the algorithm diverges into two parts, namely, exploration and exploitation.

#### Exploration

The grey wolves (or the search agents) scan the search space according to the positions of alpha, beta and delta. They move across the search space away from each other in search of the prey and come together to attack the prey. The mathematical model of divergence or exploration is given by A with random values, if $\vec {A}>1$ the wolves diverge from the prey in order to find a better prey.

The exploration process is also taken care by the second coefficient $\vec {C}$. This coefficient gives us random values from [0, 2] as Eq. (4) depicts. $\vec {C}>1$ provides a better exploration to find a better solution than the previous. The random assigning of weights helps the algorithm with defining the distance between the prey and the wolves.

This process allows the algorithm to be more random during optimisation and also helps with avoiding local minima. To maintain this process, the $\vec {C}$ provides random values at all times so as to maintain exploration not only in the initial iterations but also the final iterations.

#### Exploitation

Once the grey wolves finish the hunting phase, they start the next phase known as exploitation (attacking). The mathematical model requires decreasing value of $\vec {a}$ from 2 to 0 during the iterations linearly, which means $\vec {A}$ is a random value in the interval [− 2a, 2a]. When $\vec {A}$ is in [− 1, 1], the next position of the wolves lies between the current position of the wolf and the position of the prey. In such a case, $\vec {A} \le 1$ is used to converge to the position of the prey, which is provided by the best three fitness solutions.

### Sequential analysis

The SA is a method that uses a priori information about the maternal peaks proposed by^20^. This information is used to detect the maternal signal and create a template using averaging and scaling methods. An accurate formation of the mECG template leads to a better mECG cancellation. The method consists of a scaling procedure, but instead of scaling the average of the whole mECG cardiac cycle $\mu$, the scaling is performed separately on the P-wave, QRS complex, and the T-wave. This is done in order to solve the time-varying morphology of the mECG that occurs due to breathing and movement. Within each mECG complex, the P-wave, QRS complex, and T-wave are isolated. The total length of the mECG window is 0.70s. The mECG window is split into the following sections:

- $\mu _R$—samples between 0.05 s before and after an R peak detected are considered a QRS complexes.
- $\mu _P$—samples before 0.20 s before the QRS complex are considered P-waves.
- $\mu _T$—samples before 0.40 s after the QRS complex are considered T-waves

The matrix with the P-wave, QRS complex and T-wave vectors is defined as:

9 $$\begin{aligned} M = \begin{pmatrix} \vert &{} 0 &{} 0\\ \mu _p &{} 0 &{} 0\\ \vert &{} 0 &{} 0\\ 0 &{} \vert &{} 0\\ 0 &{} \mu _{QRS} &{} 0\\ 0 &{} \vert &{} 0\\ 0 &{} 0 &{} \vert \\ 0 &{} 0 &{} \mu _T\\ 0 &{} 0 &{} \vert \end{pmatrix}. \end{aligned}$$

The mECG complex template, ${\hat{m}}$ is given by ${\hat{m}}=Ma$, where *a* is a scaling vector, $a=$ ($a_P$ $a_{QRS}$ $a_T$). The value of *a* for each vector is given by:

10 $$\begin{aligned} {a}=\left( M^{T} M\right) ^{-1} M^{T} m. \end{aligned}$$

The scaling is done in order to get the LMS $e^2$.

11 $$\begin{aligned} {e^2}=min|\mu a-m |^2. \end{aligned}$$

After the construction of the maternal template, it is then used to cancel mECG. A QRS detector is used to detect the fetal peaks.

When using the SA method, the scaling factor values used to create the matrix are crucial to building a maternal template that adapts to each maternal peak. These templates lead to the elimination of the maternal peaks in the signal. The elimination of the maternal component is only as good as the created template. The approach provided here adapts to the varying nature of the signal using the scaling vector that forms the matrix. The value of the scaling vector for each peak influences the accuracy of the template. To achieve the desired accuracy, we propose, SA combined with GWO to generate a maternal template effectively.

### Datasets

In this study, we used signals from two real datasets available on a public server, and were recorded under clinical conditions as part of research projects at the Department of Obstetrics and Gynecology of the Medical University of Silesia in Katowice, Poland. Research was approved by the University’s Bioethics Committee (Commission approval number NN-013-345/02). The subjects read the approval consent form and gave a written consent to participate in the study. The datasets analysed during the current study are available in the figshare repository integrated with Scientific Data Journal, detailed information could be found in^18^.

The aECG signals in both datasets from the above mentioned public domain were recorded from the maternal abdomen using the KOMPOREL system. The sensing electrodes were placed around the maternal navel line, a common reference electrode was placed over the pubic symphysis, and a reference electrode was placed on the maternal left leg. The direct fECG signal was recorded from the fetal head using a sterile spiral electrode. The signals were digitized with a 16-bit resolution and a sample frequency of 500 Hz for aECG signals and 1000 Hz for direct fECG signals.

The information regarding both of the datasets is summarized in Table 1. The Labour dataset contains 12 records of 5 min from women in advanced pregnancy between 38 and 42 weeks of pregnancy. Each record contains 4 aECG signals, and the record also includes a direct fECG signal simultaneously recorded from the head of the fetus using the scalp electrode. The Pregnancy dataset contains 10 records of 20 min from women between 32 and 42 weeks of pregnancy. Each record also contains 4 aECG signals, but in this case, no direct reference fECG has been recorded. Both datasets contain annotations with the exact positions of the fQRS complexes determined by the automatic detection of R-peaks. The accuracy of the fQRS positions determined was confirmed by clinical experts. Unfortunately, the dataset does not include further information regarding the tested subjects which prevents further tests of clinical dependency of the methods with the anamnestic data of involved subjects.

**Table 1:** Summary of the datasets used for the experiments.

	In labour	Subjects	Week of pregnancy	Length (min)	Length (samples)
Labour	Yes	12	38–42	5 × 4 × 12	150,000 × 4 × 12
Pregnancy	No	10	32–42	20 × 4 × 10	598,900 × 4 × 10
Summary	–	22	32–42	1040	31,156,000

### Evaluation protocols

Contrary to other fields, it is not possible to simply use *objective* metrics such as SNR, RMSE, and others. This kind of assessment is only possible when the *synthetic* data is used, where the outcomes often do not correspond to those obtained in experiments with *real* signals. The main reason is that the *ideal* fECG signal is not available in case of real signals. The only signal that can be obtained in the case of fECG measurement is the *direct* fECG acquired using the fetal scalp electrode (FSE). However, this signal does not fully correspond to the fetal component in the aECG signal due to the dispersion caused by the signal propagating from the fetal to the maternal body, which results in morphological changes of the abdominal fECG component. Therefore, the FSE signal is only acceptable as reference (so-called *silver standard*) for the fHR-based assessment but not to fully assess the morphology of the signal^1^.

The approach in evaluation of the results in fECG extraction is thus not a simple task. In this study, we included different methods that are either prevalent in the literature or clinically relevant for the diagnostic purposes. The evaluation protocol consisted of three parts that aimed at assessing the algorithm’s ability to recover the fECG signal from the composite abdominal mixture. These three main parts differed both in the parameters used for the assessment and the purpose they were selected for as described below:

1. *Evaluation of the R-peak detection accuracy*—for this purpose, we used a commonly used objective evaluation metrics defined in the following subsection. These metrics are commonly used in the field of fECG extraction and thus allow for the comparison with other state-of-the-art methods.
2. *Evaluation of the clinically important features*—the clinical features derived from the extracted signals are more important for the clinical evaluation of the R-peak identification and thus they are more suitable to demonstrate the clinical use of the method.
3. *Evaluation using the signal quality indices*—this is an additional parameter assessing the overall quality of the output signal.

#### Evaluation 1: R-peak detection accuracy

Determination of this parameter is used in various publications focused on fECG signal extraction and determination of R-peak positions, such as^21,22^. To calculate the selected parameters, the values of the peaks detected in the extracted signals were located and compared with the reference annotations. Based on that, these peaks were categorized as the true positive (TP), false positive (FP), or false negative (FN). The TP peaks are the R-peaks in the extracted signal, which lie within ± 50 ms interval from the reference annotations. Detected R-peaks in the extracted signal, which fall outside the mentioned interval, are determined as FP. Finally, the omitted R-peaks are determined as FN, which were to be detected in the mentioned interval, but were missing there. After determining these parameters (TP, FP, and FN), it is possible to calculate following objective evaluation parameters: accuracy (ACC), sensitivity (SE), positive predictive value (PPV), and the F1 score (a harmonic mean of the SE and PPV) using Eqs. (12)–(15), respectively^23^.

12 $$\begin{aligned} ACC= & {} \frac{TP}{TP+FP+FN} \cdot 100 { (\%)}. \end{aligned}$$

13 $$\begin{aligned} SE= & {} \frac{TP}{TP+FN} \cdot 100 { (\%)}. \end{aligned}$$

14 $$\begin{aligned} PPV= & {} \frac{TP}{TP+FP} \cdot 100 { (\%)}. \end{aligned}$$

15 $$\begin{aligned} F1= & {} 2 \cdot \frac{SE \cdot PPV}{SE+PPV} \cdot 100 { (\%)}. \end{aligned}$$

#### Evaluation 2: clinically important features

Moreover, besides the statistical metrics evaluating the accuracy of the R-peak identification, we also computed the R-peak-derived clinical features to demonstrate the clinical usability of the method. To compare the values obtained from the extracted signal, we used the clinical parameters provided by the authors of the databases used (see^24^), namely the *Basal fHR* and *fHR fluctuations*, which does not correspond to the official nomenclature. Additionally, it is unclear how these values were obtained, the authors only provide the average values summarized in a table. Our values were obtained according to the definitions of fHR characteristics and patterns proposed by the National Institute of Child Health and Human Development (NICHD)^25^:

- *Baseline rate*—the mean bpm (rounded to 0 or 5) over a 10-min interval, excluding periodic changes, periods of marked variability, and segments that differ by more than 25 bpm.
- *Variability*—the fluctuations in baseline that are irregular in amplitude and frequency. These fluctuations are visually quantitated as the amplitude of the peak to trough in BPM.

For the sake of clearness in terms of the nomenclature, we note that the *Baseline rate* and *Variability* correspond to the *Basal fHR* and *fHR fluctuations* in^24^, respectively.

Finally, the accuracy of the results was also assessed visually using the main parameter used in the clinical practice—the fHR traces. Thus, to demonstrate the clinical feasibility of the fECG technique and the proposed extraction system, we depicted the fHR traces determined using the extracted signals along with the fHR traces obtained from reference annotations. To plot both estimated and reference fHR traces, it was necessary to determine the beat-to-beat fHR (using the interval between the individual R-peaks) and to use a moving average with a window length of 30 samples.

#### Evaluation 3: signal quality indices

There is a variety of SQI methods in the ECG domain differing in their category. The SQI methods can be categorized as time or frequency based, detection based or fECG specific approaches. Amongst those metrics are adaptations of adult ECG SQI algorithms. The SQI methods also differ in terms of their requirement of input channels (single channel or multichannel methods). For the purpose of this study, we included two metrics also included in^26,27^: sSQI and kSQI (skewness and kurtosis, respectively).

## Sequential analysis with grey Wolf optimisation

In this section, the proposed process of SA with GWO (SA-GWO) algorithm is provided.

**Figure 2:**
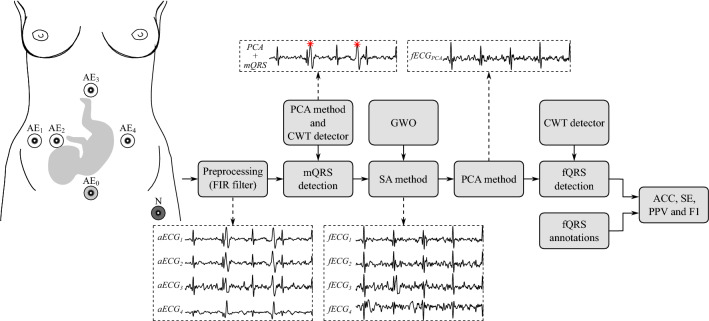
Block scheme of proposed experiment.

**Figure 3:**
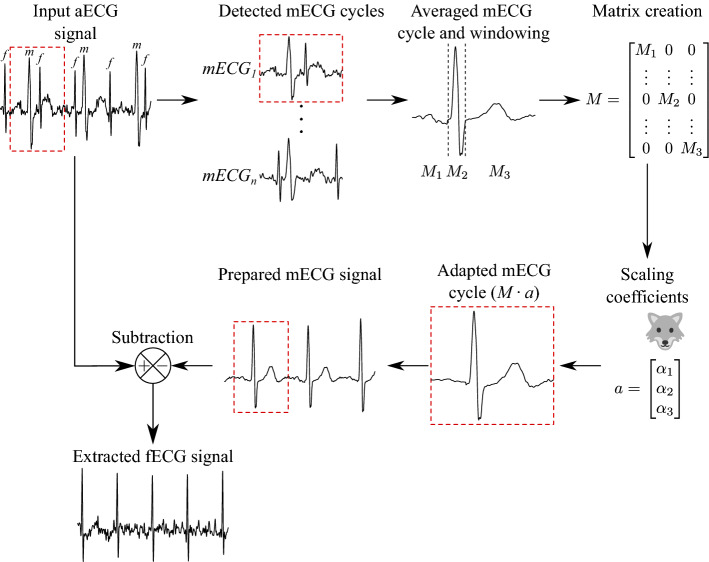
Block scheme of the SA with GWO.

The proposed algorithm is a combination of the GWO and the SA method. Figure 2 illustrates the process from obtaining the signals to finally extracting the fECG. A closer look also reveals the positioning of the abdominal electrodes ($AE_1, AE_2, AE_3,AE_4$) that were used to obtain the input signals. The input signals (abdominal ECGs) first go through pre-processing, which removes baseline wander and power-line interference from the input signals. The next stage is to detect the maternal QRS peaks to create templates that would match the individual parts of the mECG cycle: P wave, QRS complex, and T wave, leading to their elimination in the next stage. The PCA method is used to select the principle component from the abdominal signals corresponding to mECG. Following stage is the integration of SA with GWO, which would provide with the optimal matrix values to create a template that matches the time-varying morphology of the mECG signal. The process of SA with GWO is further illustrated in Fig. 3. The PCA method is used again to enhance the fECG component in the estimated signal to further improve the fHR detection. The quality of the detected fECG has to pass the statistical analysis, which is described in the following section.

Figure 3 illustrates the intake of the input signal, the detection of maternal peaks, and thereafter the process of the fECG extraction. The SA method averages all the mECG cycles and averages them. The windowing process separates the three sections of the maternal cycle, which are: P-wave, QRS-complex, and T-wave; a matrix is created to store these values. As we know, each maternal cycle has a time-varying morphology. The next step, known as scaling, is performed to adapt each created cycle to the original cycles in the input signal. The values given by the scaling vector is crucial to gain the adapted mECG cycle. Herein, we used the GWO algorithm to optimise these values in order to gain a better scaling vector for the particular input signal. Once the signal is adapted to the particular mECG cycle, it is eliminated from the original signal. This process is repeated for each detected mECG cycle, hence, leaving with the fECG signals. Once extracted, these fECG signal is sent for further quality assessment as shown in Fig. 2.

The reason to integrate SA-GWO is to obtain an optimal value of the scaling vector, *a*, described in the previous section. Three optimal values are selected for the three sections of the maternal cycle based on the updated position of the search agents.

GWO algorithm within the SA functions in the following manner:

**step 1:** The search agents are initialised by assigning random position value of the agents for the scaling vector, *a*.

**step 2:** The grey wolf optimiser runs for each detected mECG to give the three optimal scaling factors, which is obtained from the alpha position for individual parts of the mECG cycle: P wave, QRS complex, and T wave.

**step 3:** The newly created template is then used to eliminate the maternal component in the input signal.

**step 4:** The grey wolf optimiser is given the following objective to minimise:

16 $$\begin{aligned} J= & {} \min \left( \sum _{n=1}^{N}(input~signal-mECG ~template)^{2}\right) \end{aligned}$$

17 $$\begin{aligned} J= & {} MSE(input~signal-mECG ~template) \end{aligned}$$

Increasing the number of iterations and the number of search agents increases the complexity but can give a slightly higher accuracy at the cost of an increase in computational time. The computational complexity of the algorithm in such cases can be addressed by using the processing speed of a field-programmable gate array (FPGA), which would improve both time and power consumption^28^. Though we have selected GWO for the purpose of extracting fECG, we cannot conclude that GWO is the only optimiser fit to perform the task discussed in the paper. The results are further discussed in the following section.

## Results

This section shows the results of the experiments on real data assessed using the evaluation protocols defined above.

The results for the parameters ACC, SE, PPV and F1 are summarized in Table 2 for both the Labour and the Pregnancy dataset. For the Labour dataset, according to the ACC parameters, a highly accurate extraction was achieved, i.e. a value higher than $95.00\%$, for all records except of record r03. For record r03, the value of the ACC parameter was low ($58.13\%$), which was caused by a high number of FN and FP values (169 and 225, respectively). Such a low extraction accuracy could probably lead to an inaccurate diagnosis of the fetal health state. For the Pregnancy dataset, the ACC values were lower than $95.00\%$ for records r02, r06, r07 and r10, but in this case the drop in accuracy was not so significant (in all four cases the ACC values were $\ge 88.00\%$), which should not affect the resulting diagnosis of the fetal hypoxia. Regarding SE, PPV and F1 parameters, values higher than $95.00\%$ were achieved for all three parameters for all records except r03 (SE = $76.40\%$, PPV = $70.85\%$, F1 = $73.52\%$) for the Labour dataset and for all records except r10 for the Pregnancy dataset. Additionally, no FP or FN values were detected for record r10 from the Labour dataset, and all four parameters reached the values of $100.00\%$. Similarly, the r08 record from the same dataset detected no FP values and only one FN value, resulting in a PPV of $100.00\%$.

Since the proposed algorithm is of stochastic nature, we ran the algorithm multiple times to record the minimum accuracy average and maximum accuracy average. We conducted 10 independent runs of the algorithm for each combination in both datasets to assure its repeatability under the same conditions. For the Labour dataset, the minimum accuracy achieved from the average of all the signal combinations was $94.07\%$, and the maximum accuracy achieved from the average of all the signal combinations was $94.60\%$. As for the other parameters, we obtained the average values as follows: F1 of 96.82%, SE of 97.49%, and PPV of 98.96%. The similar test was performed on the Pregnancy dataset. The minimum accuracy achieved from the average of all the signal combinations was $94.88\%$ and the maximum accuracy achieved from the average of all the signal combinations was $95.66\%$. For the rest of the parameters, following average values were achieved: F1 of 97.44%, SE of 98.07%, and PPV of 97.44%.

The reference and estimated values of the clinically significant parameters of baseline fHR and fHR variation were determined for both databases and summarized in Table 2. The values of baseline fHR estimated from the extracted fECG signals in the Labour dataset deviated most from the reference value in record r03 (the difference was 23.02 bpm), in record r10 with a difference of 6.54 bpm and for recording r11 with a difference of 7.37 bpm. In the Pregnancy dataset, the biggest deviation was observed in the record r04 with a difference of 8.46 bpm and in record r03 with a difference of 5.68 bpm. For the other recordings, the deviation values were lower than 5 bpm and can therefore be considered negligible. The lowest difference was achieved in the Labour dataset in the case of the record r01 with a difference of only 0.42 bpm and in the Pregnancy dataset with record r05 with even lower difference of 0.32 bpm. For fHR fluctuations, the largest deviation in the Labour dataset was observed in the case of record r04 with a value of 6.40 bpm, followed by a deviation in the record r01 with a value of 6.37 bpm and a deviation in the record r03 with a value of 5.42 bpm. For the rest of the recordings, as well as for all the recordings from the Pregnancy dataset, the difference values were lower than 5 bpm and can also be considered negligible. The lowest difference was achieved for the Labour dataset at record r08 with a value of 0.70 bpm and for the Pregnancy dataset at record r06 with a value of 0.4 bpm.

The results of kSQI and sSQI for both databases are summarized in Table 3. As these are parameters that are used to evaluate mainly adult ECG, there are no established threshold values or ranges that could be used for fECG evaluation. For these reasons, we will use the knowledge that is used in the adult ECG. According to^29^, the higher the value of the kSQI and sSQI indices, the fewer outliers equivalent to noise the signal contains, and thus the signal is of better quality. For kSQI, if its value is higher than 5, the signal is of high quality. According to the results in Table 3, the values of kSQI $>5$ were achieved for all recordings in both datasets, and according to this parameter, all extracted signals were of high quality. According to kSQI, the highest value, and thus the best extraction, was achieved for the Labour dataset with the r07 record with a value of 21.12 and for the Pregnancy dataset with the r04 record with a value of 55.09. Conversely, according to kSQI, the lowest quality extraction was achieved for the Labour dataset with record r03 with a value of 7.73 and for the Pregnancy dataset with record r07 with a value of 8.80. Regarding the sSQI, the best result was achieved for the Labour dataset with the r08 record with a value of 1.19 and for the Pregnancy dataset also with the r08 record with a value of 6.33. The worst results were achieved for the Labour dataset with the r10 record with a value of − 1.94 and for the Pregnancy dataset also with the r10 record with a value of − 1.82.

**Table 2:** Evaluation parameters of the R-peak detection accuracy obtained by SA-GWO algorithm tested on signals from labour dataset and the pregnancy dataset.

Dataset	Recording	Parameters						
		TP (–)	FP (–)	FN (–)	ACC (%)	SE (%)	PPV (%)	F1 (%)
Labour	r01	640	5	4	98.61	99.38	99.22	99.30
	r02	612	5	25	95.33	96.08	99.19	97.61
	r03	547	225	169	58.13	76.40	70.85	73.52
	r04	671	13	10	96.69	98.53	98.10	98.32
	r05	653	7	7	97.90	98.94	98.94	98.94
	r06	676	6	8	97.97	98.83	99.12	98.98
	r07	619	10	13	96.42	97.94	98.41	98.18
	r08	644	0	1	99.84	99.84	100.00	99.92
	r09	666	9	8	97.51	98.81	98.67	98.74
	r10	627	0	0	100.00	100.00	100.00	100.00
	r11	640	10	6	97.56	99.07	98.46	98.77
	r12	655	3	2	99.24	99.70	99.54	99.62
Pregnancy	r01	3101	19	17	98.85	99.45	99.39	99.42
	r02	2684	51	107	94.44	96.17	98.14	97.14
	r03	2461	21	96	95.46	96.25	99.15	97.68
	r04	2760	6	14	99.28	99.50	99.78	99.64
	r05	2762	7	2	99.68	99.93	99.75	99.84
	r06	2814	102	65	94.40	97.74	96.50	97.12
	r07	2962	117	134	92.19	95.67	96.20	95.94
	r08	2864	64	33	96.72	98.86	97.81	98.33
	r09	2787	39	29	97.62	98.97	98.62	98.79
	r10	2427	175	156	88.00	93.96	93.27	93.62

**Table 3:** Result of Basal fHR, fHR fluctuations, Kurtosis and skewness analysis for the labour dataset and the pregnancy dataset.

Dataset	Recording	Baseline rate		Variability		kSQI (–)	sSQI (–)
		Ref. (bpm)	Est. (bpm)	Ref. (bpm)	Est. (bpm)		
Labour	r01	129.04	129.46	7.20	13.57	10.58	1.12
	r02	133.69	132.91	7.60	9.17	12.76	1.13
	r03	148.83	171.85	14.70	20.12	7.73	− 0.30
	r04	137.42	141.34	9.20	15.60	11.75	− 0.48
	r05	133.05	134.00	6.90	8.80	15.45	− 1.52
	r06	136.92	138.70	9.50	8.20	12.11	− 1.01
	r07	126.88	126.09	6.40	8.30	21.12	− 1.47
	r08	129.09	133.26	7.20	6.50	12.83	1.19
	r09	134.93	138.26	9.00	4.60	9.93	− 0.85
	r10	125.47	132.01	6.70	8.30	16.15	− 1.94
	r11	130.40	137.77	7.10	8.70	15.75	0.88
	r12	131.47	132.92	8.10	6.40	11.53	0.84
Pregnancy	r01	156.56	157.53	14.10	12.80	13.56	− 1.62
	r02	140.58	136.45	11.20	15.83	13.00	− 1.81
	r03	128.92	123.24	7.20	7.70	17.08	1.79
	r04	139.47	147.93	11.70	14.90	55.09	− 1.40
	r05	138.88	139.20	10.50	9.40	15.63	1.99
	r06	144.70	147.05	11.30	10.90	50.53	− 1.16
	r07	156.04	157.70	15.41	11.40	8.80	− 0.71
	r08	145.91	146.59	12.50	16.60	14.41	6.33
	r09	143.44	145.38	11.60	9.20	37.38	0.97
	r10	131.60	133.46	10.00	13.90	14.53	− 1.82

## Discussion

**Figure 4:**
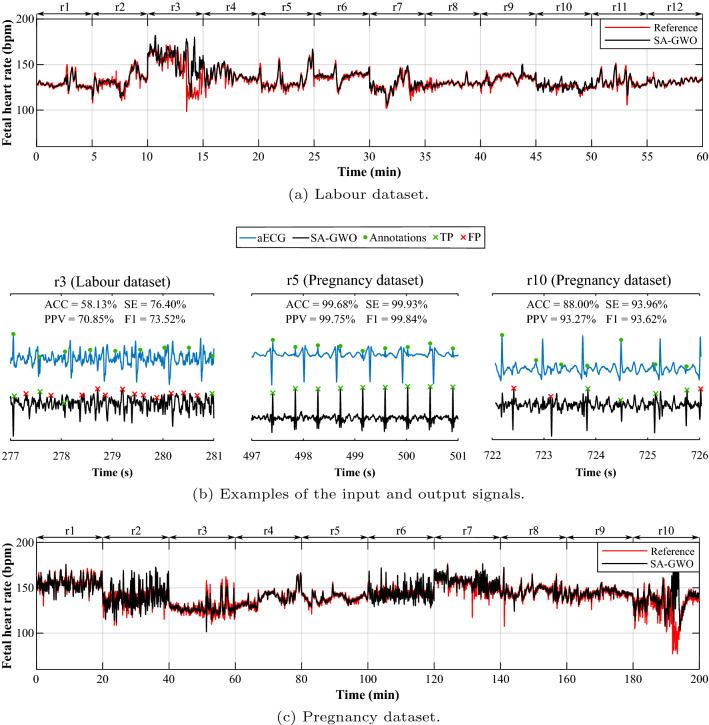
Comparison of fHR traces determined from signals extracted with the SA-GWO method and reference fHR trace for all recordings and the examples of the signals from selected records: (**a**) the fHR trace determined from the Labour dataset records using the fECG signal extracted by the SA-GWO system (black) in comparison with the reference fHR trace (red); (**b**) examples of the extracted signals from records achieving low (r3, r10) and high (r5) quality results along with the obtained parameters and illustration of TP and FP values; (**c**) the fHR trace determined from the Pregnancy dataset records using the fECG signal extracted by the SA-GWO system (black) in comparison with the reference fHR trace (red).

The results presented in the previous section demonstrated high effectiveness of the proposed system in extracting the fECG signal. In the clinical practice, the fHR traces are monitored and visually assessed during the pregnancy and the labor to assess fetal health state. To demonstrate the applicability of the fECG technique and the proposed extraction system in the clinical practice, we plotted the fHR traces estimated from abdominal ECG records along with the fHR traces obtained from reference annotations. The resulting fHR traces for all records from the Labour and the Pregnancy dataset are shown in Fig. 4a,c, respectively. In the case of the Labour dataset, the estimated fHR traces copy the trend of the reference fHR trace for all records except record r3. In the case of the Pregnancy dataset, the estimated fHR traces copy the trend of the reference fHR trace for all records except for record r10. This corresponds to the above results—these two records achieved the poorest results in most of the tested parameters.

To investigate the reasons why some of the outputs were not as accurate as others, we carried out a detailed analysis of the extracted data. For this investigation we selected samples of the signals from recordings that achieved poor results (r3 form Labour dataset and r10 from Pregnancy dataset) and the signals from recordings associated with high accuracy (r5, Pregnancy dataset). In both cases, we plotted the input aECG signal with annotations and the SA-GWO output. One can notice that the input signals from recordings r3 and r10 were both of poor quality. They are either too noisy or the ratio between the maternal and fetal component was too low. Both led to the inability of the algorithm to extract fECG signal of sufficient quality. For r3 recording, the fetal peaks were hidden in the noise, which was of the same amplitude and thus they were incorrectly detected—this led to high amount of falsely detected peaks and thus higher fHR than the one in the reference signal. This is prominent also in the lower values of objective parameters (ACC = $58.13\%$, SE = $76.40\%$, PPV = $70.85\%$, F1 = $73.52\%$). In r10 recording, the output signal contained maternal residue of amplitude comparable with the fetal peaks. These were falsely detected as fetal R-peaks and, similarly as in the previous case, led to higher fHR in the resulting trace. Again, this resulted in decreased values of the evaluation parameters (ACC = $88.00\%$, SE = $93.96\%$, PPV = $93.27\%$, F1 = $93.62\%$) in comparison with the remaining records (except r03, which besides the maternal residue also contained significant amount of noise). In contrast, when inspecting the signals on the examples from the r5 recording, where the extracted signals are of high quality, the maternal component was entirely suppressed and there is no additional noise. This is thanks to sufficient quality of the aECG input, which is not affected by any high frequency noise and the ratio between fetal and maternal component is high.

It is important to note that although the system achieved high accuracy when evaluated using statistical parameters (see Tables 2, 3), the method failed in some instances when comparing the obtained fHR trace with the reference one (see Fig. 4). In the cases when the quality of the signal input was low, the output fHR trace deviated from the reference one—it was mostly higher. That would be a real problem if this method is used in clinical practice since the fHR trace is the main parameter to assess fetal well-being. When the fHR is low, it is often associated with fetal hypoxia, whereas higher fHR may be caused with increased fetal mobility. Therefore, such inaccuracy in fHR determination could lead to misdiagnosis of fetal health state. Future research should also include a system for evaluation of the input signal quality used for selection of suitable electrodes for the signal acquisition. This would involve evaluation of several factors including presence of noise (caused by e.g. myopotentials or external devices), fetal position or gestational age (influencing the ratio between maternal and fetal component in the aECG signal).

Finally, we compared the results with those achieved by other authors in the last 2 years, see Table 4. Objective comparison of results is challenging due to the different methodology applied in each study. The authors use different types of signals (real or simulated^30,31^). Moreover, these signals come from different databases, e.g. *the Fetal ECG Synthetic Database (FECGSYNDB)*^32^, *the Non-Invasive fECG Database (NIFECGDB)*^33^, *Computing in Cardiology Challenge 2013*^34^ or *Abdominal and Direct Fetal ECG Database (ADFECGDB)*^35^. The evaluation of the extraction quality is not uniform either, as various parameters are used, e.g. ACC, F1 or signal-to-noise ratio improvement (SNR$_{\textrm{imp}}$). Therefore, although one selects the results that were tested on same databases (Challenge 2013 and ADFECGDB), the data used for the verification may differ. For example, in case of Challenge 2013 database, some authors use all 75 records, whereas some used only part of them. This dataset includes both real and synthetic data which are not differentiated. Therefore, such dataset does not reflect the method’s applicability in the clinical practice. For this reason, we decided not to include this dataset in our evaluation.

Another key point to address is the time complexity of the algorithm. The GWO convergence time highly depends on the parameters such as the number of iteration as well as the number of search agents. The higher number of iterations and search agents do not provide a significant change in the accuracy of the results. Also, the GWO is run within the SA to adapt to each maternal template. This also rises the computational time significantly, in contrast to a non-adaptive algorithm, which does not adapt according to each template, but takes an average of the whole maternal component. The parameters were selected keeping these aspects in mind. An addition to the proposed method would be to use a FPGA that would impact the speed and power at which the computation is performed.

In case of ADFECGDB, there are differences between the authors too. This is caused by the fact that the database initially contained 5 recordings (r01, r04, r07, r08, and r10). However, some authors^6^ also tested the remaining recordings (r01–r12) that were published later in^24^, named Labour dataset. Therefore Tab. 4 includes both differentiated as “ADFECGDB” and “Labour”, respectively.

**Table 4:** Comparison of results with the latest studies focused on fECG extraction.

Author, source, year	Algorithm	Dataset	Result
Castillo et al.^36^, 2018	WT-CT	ADFECGDB	ACC = 88.87%
		Challenge 2013	ACC = 96.21%
Zhong et al.^37^, 2019	RCED-Net	ADFECGDB	F1 = 94.10%
		Challenge 2013	F1 = 93.62%
Jaros et al.^7^, 2019	ICA-RLS-WT	ADFECGDB	ACC = 85.92%
		Challenge 2013	F1 = 68.25%
Gurve et al.^38^, 2020	CS-NMF	ADFECGDB	F1 = 94.80%
		Challenge 2013	F1 = 84.00%
Taha et al.^39^, 2020	NSITM	Challenge 2013	ACC = 97.00%
Zhang et al.^30^, 2020	K-means-PCA	ADFECGDB	F1= 96.09%
		Challenge 2013	F1 = 95.47%
Krupa et al.^40^, 2021	FrFT-WT	Challenge 2013	ACC = 92.68%
		Own real dataset	ACC = 96.98%
Jallouli et al.^41^, 2021	WT-entropy	Challenge 2013	ACC = 98.96%
Barnova et al.^6^, 2021	ICA-FTF-CEEMDAN	Labour dataset	ACC = 92.98%
		Challenge 2013	ACC = 78.47%
Kahankova et al.^8^, 2022	ICA-ADALINE	Labour dataset	F1 = 94.06%
		Challenge 2013	F1 = 92.47%
Proposed algorithm	SA-GWO	Labour dataset	ACC = 94.60%
		Pregnancy dataset	ACC = 95.66%
		ADFECGDB	F1 = 98.77%
			ACC = 97.58%

Following studies were used for the comparison:

- A single-channel method combining compressive sensing (CS) and non-negative matrix factorization (NMF) was proposed by Gurve et al.^38^. The algorithm was tested on real records from the ADFECGDB and Challenge 2013 dataset, where it reached F1 = 94.80% and F1 = 84.00%, respectively. In addition, the method has proven to be cost-effective and therefore suitable for implementation within a battery-powered remote monitoring device.
- Research of Taha et al.^39^ introduced a blind source separation-based method, and a new null space idempotent transformation matrix (NSITM) algorithm. When testing the method on signals from the Challenge 2013 dataset, an average value of ACC = 97.00% was achieved.
- Zhang et al.^30^ proposed a combination of k-means and PCA methods for fECG extraction. Fetal and maternal QRS complexes were detected by k-means clustering and subsequently the resulting fECG signal was extracted using a PCA-based subtraction template. The performance of the method was verified on real records from the ADFECGDB and Challenge 2013 datasets. High accuracy was achieved in both databases F1 = 96.09% and F1 = 95.47%, respectively.
- Krupa et al.^40^ proposed fECG extraction in the time-frequency domain using a combination of fractional Fourier transform (FrFT) and WT. The parent component was suppressed by FrFT and interference balances were removed by WT. When testing on records from Challenge 2013 dataset, ACC = 92.68% was achieved and when testing on own real records ACC = 96.98%.
- Jallouli et al.^41^ used the Clifford wavelets-based WT method for fECG extraction combined with Shannon entropy. When tested on Challenge data, ACC = 98.96% was achieved with the Clifford wavelets application, which exceeded the performance of the Haar–Faber–Schauder wavelets.
- Algorithm combining independent component analysis (ICA), adaptive fast transversal filter (FTF) and complementary ensemble empirical mode decomposition with adaptive noise (CEEMDAN) was presented by Barnova et al.^6^. The method was tested on real records from Labour datasets, for which efficient extraction was achieved (ACC = 92.98%) and on records from Challenge 2013 datasets, for which the method was less accurate (ACC = 78.47%) due to lower quality input aECG signals. In addition, non-invasive ST analysis of the extracted fECG signals was successfully performed.
- The combination of wavelet transform (WT) and clustering-based technique (CT) was presented by Castillo et al.^36^. The experiments performed on ADFECGDB dataset and Challenge 2013 dataset resulted in ACC= 88.87% and ACC= 96.21%, respectively. However, the authors did not use all of the recordings from the dataset as they excluded records of lower quality.
- Zhong et al.^37^ proposed a deep learning technique based on residual convolutional encoder-decoder network (RCED-Net) for fECG extraction. This model was trained on signals from the Fetal ECG Synthetic Database (FECGSYNDB) and signals from the ADFECGDB and Challenge 2013 dataset were used for testing. When testing on records from the ADFECGDB dataset, the average value of F1 = 94.10% was reached and when testing at Challenge 2013, the value of F1 = 93.62% was achieved. The advantage of this approach was that it was only a single-channel method. The proposed method could extract fECG directly from the aECG without cancelling the mECG waveform
- In^7^, Jaros et al. introduced and tested a hybrid method combining the ICA algorithm, the adaptive recursive least squares (RLS) algorithm and the WT. The method was tested on the ADFECGDB and Challenge 2013 datasets, achieving ACC = 85.92% and ACC = 68.25%, respectively. The application of the WT method in the post-processing phase led to an overall improvement in extraction, but at the same time, the signal morphology was affected. This would not allow accurate morphological analysis of the signal in the future. The authors further stated that extraction was not successful for low quality of the aECG signals.
- In^8^, Kahankova et al. tested and compared several combinations of LMS and RLS based adaptive algorithms with the ICA method and proposed an optimization scheme to increase their efficacy. The most accurate combination turned out to be one with the adaptive linear neuron (ADALINE), i.e. ICA-ADALINE hybrid extraction method achieving an average value of F1 = 94.06% and 92.47% for ADFECGDB and Challenge 2013 datasets, respectively.

We can conclude that our solution outperforms the ICA-FTF-CEEMDAN solution in Labour dataset. As for ADFECGDB, the algorithm provides better outcomes in comparison with all of the tested methods.

In this paper, we focused on improving the extraction of the fetal component from the composite abdominal signal, however, there is still a space for further improvement and inclusion of more advanced methods to increase its accuracy but also extend the applicability of the system (e.g. on extracting more features from the signal or classifying the fetal health state). The latest research focus is on the implementation of machine learning (ML) methods into given field. Nevertheless, up to date, there have been only a few authors that focused on the fetal extraction using ML^42–44^, mostly on synthetic or own data. The ML methods are more used in the area of fetal health state classification based on the fHR traces obtained by the conventional CTG method. For these purposes, there are already datasets available^45,46^, and thus more research has been done there.

Preparation of a ML-suited dataset is needed if any ML methods are to be used in the future. Such dataset should be further annotated to be able to assess also the True Negative (TN) peaks. The dataset annotation would have to be prepared in a different way; no standard method to identify such peaks had been defined so far and no ML focused dataset has been introduced for the fECG extraction yet^42^.

## Conclusion

We presented a novel algorithm for fECG extraction. The GWO algorithm is combined with SA to extract the fetal from the abdominal signal by eliminating the maternal component using a maternal template. The GWO optimises the scaling vector needed to adapt the maternal template to the time varying morphology of the ECG signal. The results have shown to provide excellent accuracy with the maximum iteration as well as the number of agents of 10, respectively. The algorithm was tested on real records and its performance was objectively evaluated using the accuracy of R-peaks detection (by determining ACC, SE, PPV and F1 parameters), by evaluating clinically important features (baseline rate and variability) and by determining sSQI and kSQI indices. Although the average accuracy of the algorithm was high (ACC = 94.60% and 95.66% for the Labour and the Pregnancy datasets, respectively), a significant decrease in accuracy was observed for records r3 (Labour) and r10 (Pregnancy). These results corresponded with the results of the other parameters, since for most of them, these two records also achieved the poorest results. These findings obtained through objective evaluation were also confirmed by visual comparison of fHR traces. The visual comparison of the fHR traces was carried out since the physicians are accustomed to it in the conventional CTG monitoring and it is thus the most important parameter in clinical practice. The results showe that fHR traces were determined accurately for almost all records. Deviations of the estimated fHR trace from the reference fHR trace were significant only for r3 and r10 records, which was due to poorer quality of aECG records leading to less efficient extraction and detection of fQRS complexes. This aspect should be considered in the future research to ensure clinical applicability of the proposed system. Even though the proposed algorithm provides high accuracy, it still suffers from computational complexity. This problem can be addressed by using the processing speed of an FPGA, which would improve both time and power consumption.

## Data Availability

The datasets analysed during the current study are available on a server at the generalist repositories (figshare) integrated with Scientific Data Journal. The commission approval number is: NN-013-345/02 and can be found at https://doi.org/10.6084/m9.figshare.c.4740794^18^.
